# Risk factors analysis of Mycoplasma pneumoniae infection in children and diagnostic value of IL-6 and STAT3

**DOI:** 10.1097/MD.0000000000044772

**Published:** 2025-10-24

**Authors:** Li Feng, Jingxin Mo, Junyong Zhu

**Affiliations:** a Graduate School of Jinzhou Medical University, Jinzhou, Liaoning, China; b Respiratory Intensive Care Unit, Shiyan People’s Hospital, Shiyan, Hubei, China.

**Keywords:** children, diagnostic value, interleukin-6, Mycoplasma pneumoniae, risk factors, signal transduction and transcriptional activation factor 3

## Abstract

This study analyzes the risk factors for mycoplasma pneumoniae infection in children and the expression of interleukin-6 (IL-6)/signaling and transcriptional activating factor 3 (STAT3) pathway. Seventy-three children diagnosed with mycoplasma pneumoniae infection were admitted to Jinzhou Medical University Hospital between April 2021 and April 2023, forming the study group. Additionally, 730 healthy children who underwent physical examinations during the same period were selected as the control group. A comparison of clinical data between the 2 groups was conducted to analyze the risk factors associated with mycoplasma pneumoniae infection in children. Furthermore, the diagnostic value of IL-6, STAT3, white blood cells (WBC), C-reactive protein (CRP), and procalcitonin (PCT) in children with mycoplasma pneumoniae infection was assessed using receiver operating characteristic (ROC) curve analysis. A history of recurrent upper respiratory tract infections and living in close quarters are identified as risk factors for mycoplasma pneumoniae infection in children (OR = 1.988, 2.123, *P* < .05). The study group exhibited elevated levels of IL-6, STAT3 mRNA, WBC, CRP, and PCT compared to the control group (*P* < .05). Analysis of the ROC curve revealed that using IL-6, STAT3, WBC, CRP, and PCT levels individually or in combination yielded area under the curve values of 0.829, 0.813, 0.852, 0.759, 0.849, and 0.953, respectively. The combined detection method demonstrated a sensitivity of 86.30% and specificity of 90.40%. The IL-6/STAT3 pathway is known to be activated in children with mycoplasma pneumoniae infection, leading to an upregulation in the expression of white blood cells (WBC), C-reactive protein (CRP), and procalcitonin (PCT). Utilizing a combination of IL-6, STAT3, WBC, CRP, and PCT levels can aid in the diagnosis of mycoplasma pneumoniae infection in children. Additionally, a history of recurrent upper respiratory tract infections and living in close quarters with others can increase the risk of mycoplasma pneumoniae infection in children.

## 1. Introduction

Mycoplasma pneumoniae (MP) has a pathogen size that falls between that of bacteria and viruses, and exhibits bacterial biological characteristics.^[1,2]^ Following droplet transmission, MP commonly causes upper respiratory tract infections and Mycoplasma pneumonia. While most patients experience self-limiting symptoms postinfection, a small subset of individuals with Mycoplasma pneumonia require hospitalization for treatment. In severe cases, in addition to respiratory system involvement, lesions in the central nervous system and cardiovascular system can occur, such as multiple radiculitis and myocarditis, potentially leading to serious consequences.^[3]^ MP infection is a prevalent respiratory disease in pediatric populations, typically presenting with symptoms like fever, a persistent dry cough, and intermittent fever. It is particularly common among school-age children.^[4,5]^ The incidence of mycoplasma pneumonia is on the rise annually, posing a significant threat to children’s health.^[6,7]^ Currently, clinical diagnosis of MP infection in children often relies on pathogen culture, which is time-consuming, labor-intensive, and has low sensitivity, resulting in unsatisfactory outcomes. Therefore, the identification of a rapid, accurate diagnostic method, indicator, and risk factor for diagnosing MP infection in children is of utmost importance.

Currently, the diagnosis of MP infection heavily relies on MP serological antibody testing, which unfortunately has a low positivity rate. Pathogen culture, considered the gold standard for diagnosing MP infection, is hindered by its time-consuming nature, laborious process, and low sensitivity, making it unsuitable for early clinical diagnosis.^[8]^ Procalcitonin (PCT) is a parameter utilized to monitor inflammatory diseases stemming from bacterial and other microbial infections in the human body. It is characterized by extremely low concentrations in healthy individuals and stable properties. Clinically, it is often employed for diagnosing infection types and monitoring disease severity.^[9]^ Hypersensitive C-reactive protein (hs CRP), an acute-phase response protein, is commonly used in clinical settings for diagnosing inflammatory diseases.^[10]^ The Signal Transduction and Transcription Activator 3 (STAT3) pathway can activate specific transfer factors, playing a crucial role in the body’s immune response against infections. Interleukin-6 (IL-6) primarily functions as a pro-inflammatory cytokine, triggering pro-inflammatory responses. Studies have shown that IL-6 is a versatile cytokine with a broad range of functions. During adverse conditions like infections, inflammatory cells in the body rapidly accumulate to produce IL-6, leading to an increase in its levels. Additionally, IL-6 can interact with GP130 on the cell membrane, inducing STAT3 phosphorylation and contributing to the onset and progression of infections.^[11]^

Beyond MP, the IL-6/STAT3 pathway has been implicated in a variety of other pediatric respiratory infections. For instance, in viral respiratory infections such as respiratory syncytial virus and influenza, elevated IL-6 levels and STAT3 activation are also associated with the severity of inflammation and immune response. The IL-6/STAT3 pathway plays a significant role in regulating the immune response during these infections, particularly by modulating cytokine production, inflammatory cell recruitment, and acute-phase responses. Additionally, this pathway has been linked to conditions like asthma, where persistent inflammation may involve sustained activation of IL-6 and STAT3, leading to chronic respiratory symptoms.^[11]^ Understanding the role of the IL-6/STAT3 pathway across these different respiratory infections provides further insight into its potential as a therapeutic target in pediatric respiratory diseases.

In light of this, this study aims to analyze the risk factors associated with MP infection in children and investigate changes in IL-6/STAT3 pathway expression, offering a reference point for clinical prevention strategies against MP infection in children.

## 2. Object and methods

### 2.1. Objects

A retrospective study was conducted on 730 children with MP infection admitted to Jinzhou Medical University Hospital from April 2021 to April 2023, comprising 390 males and 340 females aged 6 to 10 years. Additionally, 730 healthy children who underwent physical examinations during the same period were selected as the control group, with 400 males and 330 females aged 7 to 10 years. Inclusion criteria included diagnosis of MP infection based on the 2015 Expert Consensus on the Diagnosis and Treatment of MP pneumonia in Children, confirmed by blood routine and chest X-ray examination, absence of prior antibacterial therapy, and complete clinical data. Exclusion criteria encompassed patients with significant organ diseases, coagulation abnormalities, congenital respiratory malformations, malignant tumors, hematological and immune system diseases. The study was approved by the Medical Ethics Committee of Jinzhou Medical University (Ethics Approval No. JMUEC2021001).

### 2.2. Methods

#### 2.2.1. Data collection

The general information of all research subjects was collected through the electronic medical record system and predischarge questionnaire survey at Jinzhou Medical University Hospital. This included data on gender, age, body mass index, presence of anemia, history of recurrent upper respiratory tract infections, collective living status (defined as living in environments with frequent and close contact with others, such as boarding schools, daycare centers, or other group living settings), and malnutrition.

#### 2.2.2. Laboratory testing

Fasting venous blood was collected from enrolled research subjects in the morning. White blood cell count (WBC) was detected using a fully automated blood analyzer from Olympus. Serum C-reactive protein (CRP) and procalcitonin (PCT) were measured using the Hitachi 7600-020 fully automatic large-scale biochemical analyzer. Enzyme linked immunosorbent assay (ELISA) was employed to determine serum IL-6 levels with a kit from Wuhan Geely De Biotechnology Co., Ltd. Real time fluorescence quantitative PCR (RT qPCR) was used to assess STAT3 mRNA levels, with primer sequences synthesized by Biotechnology (Shanghai) Co., Ltd. The relative expression level of STAT3 mRNA was calculated using the 2−△△CT method.

### 2.3. Statistical analysis

The data was processed using SPSS 22.0 software (IBM Corporation, Armonk). Count data is represented as [n (%)], and compared using χ^2^ tests. Measurement data was tested for normal distribution using the K-S method, expressed as mean ± standard deviation (x ± s), and intergroup comparisons were made using *t*-tests. Risk factors for MP infection were analyzed using univariate and multivariate logistic regression methods. The combined detection method was implemented using a logistic regression model, with IL-6, STAT3, WBC, CRP, and PCT levels as variables. This model was used to assess the diagnostic value of these biomarkers in combination for MP infection. Receiver operating characteristic (ROC) curves were generated to assess the diagnostic value of IL-6 and STAT3 individually and in combination for MP infection in children. A significance level of *P* < .05 was used to determine statistical significance.

## 3. Results

### 3.1. Comparison of 2 sets of general information

A comparison of the general information between the research group (n = 730) and the control group (n = 730) revealed significant differences in several factors. The research group had a significantly higher number of children with recurrent upper respiratory tract infections compared to the control group (230 vs 110, *P* = .019). Additionally, a higher proportion of children in the research group lived in collective environments (280 vs 170, *P* = .049) and experienced malnutrition (230 vs 120, *P* = .033). However, no significant differences were observed between the 2 groups in terms of gender, age, body mass index, or anemia status (*P* > .05 for all comparisons), see Table 1.

**Table 1:** Comparison of general information between 2 groups.

General information		Research group	Control group	Statistic	*P*
		(n = 730)	(n = 730)		
Gender (example)	Male	390	400	0.028	.868
	Female	340	330		
Age (yr)		7.75 ± 1.36	8.02 ± 1.42	1.173	.243
BMI (kg/m^2^)	≥24	410	380	0.248	.618
	<24	320	350		
Anemia (cases)	Have	450	440	0.029	.865
	Nothing	280	290		
Recurrent upper respiratory tract infections (cases)	Have	230	110	5.521	.019
	Nothing	500	620		
Collective Life (cases)	Yes	280	170	3.887	.049
	No	450	560		
Malnutrition (cases)	Yes	230	120	4.547	.033
	No	500	610		

### 3.2. Univariate analysis of risk factors for MP infection

Univariate analysis revealed a statistically significant difference in the infection rate of MP in children with clinical symptoms, patchy or large dense shadows on pulmonary imaging, white blood cell count, and a history of Mycoplasma infection (all *P* < .01). Please consult Table 2 for further details. Specifically, children presenting with clinical symptoms like cough, patchy or large dense shadows on pulmonary imaging, and elevated white blood cell count showed a higher incidence of MP infection (Table 2).

**Table 2:** Univariate analysis of risk factors for Mycoplasma pneumoniae infection in children.

Variable	Number of cases	Composition ratio %	Number of cases of Mycoplasma pneumoniae infection	Infection rate (%)	*t*	*P*
Season of onset						
Spring	160	21.92	30	18.75	2.611	>0.05
Summer	100	13.69	20	17.68		
Autumn	195	26.71	38	19.49		
Winter	275	37.67	52	18.91		
Bedroom symptoms						
Cough	610	83.56	119	19.51	54.381	<0.01
Fever	524	71.78	91	17.37		
Shortness of breath	177	24.25	25	14.12		
Nasal congestion	114	15.62	16	14.04		
Other	93	12.74	13	13.98		
Chest auscultation						
Fine rales	344	47.12	66	19.19	2.768	>0.05
Phlegm purring sound	222	30.41	41	18.47		
wheezes	96	13.15	17	17.71		
Other (dry sounds, pleural friction sounds, etc)	67	9.18	10	14.93		
Pulmonary imaging shows patchy or large dense shadows						
Have	401	54.93	92	22.94	66.422	<0.01
Nothing	328	44.93	42	12.80		
White blood cell count						
Increase in height	282	38.63	57	20.21	41.286	<0.01
Normal	333	45.62	42	12.61		
Reduce	113	15.48	29	25.66		

### 3.3. Multivariate analysis of MP infection

Utilizing statistically significant indicators in univariate analysis as independent variables and the incidence of MP infection as the dependent variable, the results of multivariate logistic regression analysis indicated that a history of recurrent upper respiratory tract infections and living in a collective environment were both identified as risk factors for MP infection in children (*P* < .05), as shown in Table 3.

**Table 3:** Multivariate analysis of the occurrence of Mycoplasma pneumoniae infection in children.

Variable	β	SE	Wald	*P*	OR	95% CI
Have a history of recurrent upper respiratory tract infections	0.749	0.121	10.682	<.05	1.137	1.286–1.773
Collective life	0.701	0.037	9.004	<.05	1.213	1.679–1.681
Malnutrition	0.758	0.109	8.889	<.05	1.009	1.692–1.825
IL-6	0.607	0.183	10.984	<.05	1.835	1.282–2.628
STAT3	0.009	0.004	6.213	<.05	1.009	1.002–1.017

IL-6 = interleukin-6, STAT3 = transcriptional activating factor 3.

### 3.4. Comparison of expression levels of IL-6, STAT3 mRNA, WBC, CRP, and PCT between 2 groups

The levels of IL-6, STAT3 mRNA, WBC, CRP, and PCT in the study group were 2.01 ± 0.99, 55.21 ± 26.60 pg/mL, 11.25 ± 3.84 × 109/L, 12.28 ± 4.09 mg/L, and 1.22 ± 0.04 ng/mL, respectively. These values were found to be significantly higher than those in the control group (*P* < .05), as presented in Table 4.

**Table 4:** Comparison of IL-6, STAT3 mRNA, WBC, CRP, and PCT expression levels between 2 groups (x ± s).

Indicators	Research group	Control group	*t*	*P*
	(n = 730)	(n = 730)		
WBC (×10^9^/L)	11.25 ± 3.84	7.17 ± 2.93	8.217	<.001
CRP (mg/L)	12.28 ± 4.09	8.58 ± 2.86	6.334	<.001
PCT (ng/mL)	1.22 ± 0.04	0.75 ± 0.25	8.513	<.001
IL-6 (pg/mL)	55.21 ± 26.60	30.71 ± 13.13	28.657	<.001
STAT3 mRNA	2.01 ± 0.99	1.14 ± 0.52	6.647	<.001

CRP = C-reactive protein, IL-6 = interleukin-6, PCT = procalcitonin, STAT3 = transcriptional activating factor 3, WBC = white blood cells.

### 3.5. Logistic regression analysis of IL-6, STAT3, WBC, CRP, PCT and MP infection

Elevated levels of IL-6 and STAT3 are associated with a higher likelihood of MP infection (*P* < .05). Specifically, for each unit increase in IL-6, the probability of infection with MP increases by 1.835 times. Furthermore, the study findings suggest that IL-6 (OR = 1.835, *P* < .05) is a more effective predictor of MP infection compared to STAT3 (OR = 1.009, *P* < .05). Refer to Table 5 for more details.

**Table 5:** Logistic regression analysis of predictive factors for Mycoplasma pneumoniae infection.

Variable	β	SE	Wald	*P*	OR	95% CI
WBC	0.749	0.121	10.682	<.05	1.137	1.286–1.773
CRP	0.701	0.037	9.004	<.05	1.213	1.679–1.681
PCT	0.758	0.109	8.889	<.05	1.009	1.692–1.825
IL-6	0.607	0.183	10.984	<.05	1.835	1.282–2.628
STAT3	0.009	0.004	6.213	<.05	1.009	1.002–1.017

CRP = C-reactive protein, IL-6 = interleukin-6, PCT = procalcitonin, STAT3 = transcriptional activating factor 3, WBC = white blood cells.

### 3.6. The predictive value of individual and combined detection of IL-6, STAT3, WBC, CRP, and PCT for MP infection

The control group children were labeled as negative, while the study group children were labeled as positive. ROC curves were generated to calculate the area under the curve (AUC) for diagnosing MP infection using IL-6, STAT3, WBC, CRP, and PCT levels both individually and in combination. The AUC values were 0.829, 0.813, 0.852, 0.759, 0.849, and 0.953, respectively. The combined diagnostic approach showed a higher AUC compared to individual tests (*P* < .05), with a sensitivity of 86.30% and specificity of 90.40%, as detailed in Table 6.

**Table 6:** Prediction value of IL-6, STAT3, WBC, CRP, and PCT single and combined detection for Mycoplasma pneumoniae infection in children.

Variable	Truncation value	AUC	95% CI	Sensitivity (%)	Specificity (%)
IL-6	50.10 pg/mL	0.829	0.758–0.886	64.40	94.50
STAT3	1.55	0.813	0.740–0.873	71.20	86.30
WBC	10.22 × 10^0^/L	0.852	0.784–0.906	69.90	90.40
CRP	10.48 mg/L	0.759	0.618–0.825	68.50	79.50
PCT	1.05 ng/mL	0.849	0.780–0.903	72.60	86.30
Union	–	0.953	0.905–0.981	86.30	90.40

AUC = area under the curve, CRP = C-reactive protein, IL-6 = interleukin-6, PCT = procalcitonin, STAT3 = transcriptional activating factor 3, WBC = white blood cells.

## 4. Discussion

MP is a significant pathogen in children’s community-acquired pneumonia, with an annual infection rate ranging from 9.6% to 66.7%. It tends to be prevalent every 3 to 8 years, posing a serious threat to the lives and health of children.^[12]^ Currently, clinical diagnosis of MP infection in children relies on detecting MP serological antibodies. However, the low positive rate of this method hinders its effectiveness in aiding early clinical diagnosis.^[13–17]^ Therefore, selecting a simple and efficient method is crucial for the early diagnosis and effective treatment of MP infection in children.

The IL-6/STAT3 signaling pathway, activated by infection and other stimuli, can stimulate T and B lymphocyte proliferation and differentiation, promote synthesis of acute-phase response proteins, and trigger a cascade of inflammatory responses. This pathway is closely linked to infections within the body. IL-6, a versatile peptide cytokine, plays a crucial role in the body’s immune response and acute-phase inflammatory response. It is primarily produced by monocytes and macrophages and is integral to the development of infections. STAT3, a pivotal molecule in mediating inflammatory responses, is activated by IL-6 cytokines produced by helper T cells, ultimately contributing to infection progression.^[18,19]^ Clinical indicators such as WBC, CRP, and PCT are associated with infections. In infected individuals,^[20,21]^ WBC levels rise proportionally with the severity of infection. PCT is a procalcitonin peptide, while CRP is an acute-phase response protein. Elevated levels of PCT and CRP indicate heightened inflammation in the body, posing a significant threat to the immune system. Children with compromised immunity and limited recovery capacity are at higher risk of MP infection when WBC, CRP, and PCT levels are elevated.^[22,23]^ The study revealed higher levels of IL-6, STAT3 mRNA, WBC, CRP, and PCT in the study group compared to the control group, suggesting activation of the IL-6/STAT3 signaling pathway post MP infection. Furthermore, WBC, CRP, and PCT were upregulated. ROC curves were used to assess the diagnostic value of IL-6, STAT3, WBC, CRP, and PCT individually and in combination for detecting MP infection. The results indicated a relatively high AUC for each indicator in diagnosing the infection. This could be due to MP triggering increased production of IL-6 and STAT3, which synergistically promote the body’s inflammatory response, consequently elevating WBC, CRP, and PCT levels.

The results of the multiple logistic regression analysis in this study indicated that a history of recurrent upper respiratory tract infections and living in collective environments were identified as risk factors for MP infection in children. One possible explanation for this finding is that children with recurrent upper respiratory tract infections may have compromised respiratory antibacterial defenses, making them more vulnerable to pathogenic bacterial infections, thus increasing the likelihood of MP infection.^[24,25]^ Furthermore, children, due to their young age and limited psychological and physical resilience, may experience emotional disturbances and mental stress when separated from their parents, which could impact their immune function. Additionally, the higher concentration of individuals in collective living settings may increase the risk of MP infection.^[26]^ Based on these findings, preventive measures can be recommended. Children with a history of recurrent upper respiratory tract infections should focus on maintaining a clean living environment, ensuring good air quality, following a balanced diet, and enhancing their immune system. For children living in group settings, efforts should be made to minimize their time spent together or improve living conditions. Providing more family support for emotional and physical well-being is crucial. Proper ventilation in living spaces is also recommended to reduce the risk of MP infection.

Despite the important findings of this study, there are several limitations. Firstly, as a retrospective study, the data may be subject to recall bias and limitations in clinical records, which could affect the accuracy of the risk factors and biomarkers related to MP infection. Secondly, the study was conducted at a single institution, which may limit the generalizability of the results. Future multicenter studies will help validate these findings. Additionally, while we controlled for age and gender, other potential confounders such as comorbidities or environmental factors may not have been fully considered, which could influence the relationship between biomarkers and infection. Although the sample size is relatively large, future research should include more severe cases to better understand the diagnostic and prognostic value of these biomarkers. There may also be variability in the measurement of biomarkers due to differences in laboratory techniques and equipment. Lastly, as this study did not involve long-term follow-up, it remains unclear whether the elevated biomarkers are only associated with the acute-phase of infection or if they impact long-term health. Future research should address these limitations to further validate and refine the conclusions.

In conclusion, this study highlights the significant role of the IL-6/STAT3 signaling pathway and biomarkers such as IL-6, STAT3, WBC, CRP, and PCT in the diagnosis of MP infection in children. Our findings suggest that a combination of these biomarkers can improve diagnostic accuracy, offering a more effective approach compared to individual tests. Furthermore, a history of recurrent upper respiratory infections and living in close quarters were identified as risk factors, underscoring the importance of preventive measures for vulnerable children. However, the limitations of this study, including its retrospective design and lack of long-term follow-up, indicate the need for further research to confirm and refine these results. Future studies should focus on larger, multicenter cohorts and explore the potential long-term impacts of elevated biomarkers on children’s health.

## Author contributions

**Conceptualization:** Li Feng, Junyong Zhu.

**Data curation:** Li Feng, Junyong Zhu.

**Formal analysis:** Jingxin Mo, Junyong Zhu.

**Funding acquisition:** Junyong Zhu.

**Investigation:** Jingxin Mo, Junyong Zhu.

**Methodology:** Li Feng, Junyong Zhu.

**Writing – original draft:** Li Feng, Junyong Zhu.

**Writing – review & editing:** Li Feng, Junyong Zhu.
